# Neoadjuvant continuous infusion of weekly 5-fluorouracil and escalating doses of oxaliplatin plus concurrent radiation in locally advanced oesophageal squamous cell carcinoma: results of a phase I/II trial

**DOI:** 10.1038/sj.bjc.6604659

**Published:** 2008-09-16

**Authors:** S Lorenzen, B Brücher, F Zimmermann, H Geinitz, J Riera, T Schuster, N Roethling, H Höfler, K Ott, C Peschel, J R Siewert, M Molls, F Lordick

**Affiliations:** 1 Third Department of Internal Medicine (Haematology/Medical Oncology), Klinikum rechts der Isar, Technical University of Munich, Munich, Germany; 2 Department of Surgery, University of Tübingen, Tübingen, Germany; 3 Department of Radiation Oncology, Klinikum rechts der Isar, Technical University of Munich, Munich, Germany; 4 Department of Hematology and Oncology, University Hospital Gießen-Marburg GmbH, Marburg, Germany; 5 Institute of Medical Statistics and Epidemiology, Technical University of Munich, Munich, Germany; 6 Klinikum rechts der Isar, Technical University of Munich, Munich Center for Clinical Studies, Munich, Germany; 7 Institute of Pathology, Technical University of Munich, Munich, Germany; 8 Department of Surgery, University of Heidelberg, Heidelberg, Germany; 9 Board of Directors of the University Hospital, University of Heidelberg, Heidelberg, Germany; 10 Department of Medical Oncology, National Centre for Tumour Diseases, University of Heidelberg, Heidelberg, Germany

**Keywords:** 5-fluorouracil, chemoradiotherapy, neoadjuvant, oesophageal carcinoma, oxaliplatin, response

## Abstract

Oxaliplatin and 5-fluorouracil have a significant activity in locally advanced oesophageal squamous cell cancer (OSCC). However, their optimal dosage and efficacy when combined with concurrent radiotherapy as neoadjuvant treatment are unknown. This non-randomised, phase I/II study aimed to define the maximum tolerated dose (MTD) and assessed the histopathological tumour response rate to neoadjuvant oxaliplatin in weekly escalating doses (40, 45, 50 mg m^−2^) and continuous infusional 5-fluorouracil (CI-5FU; 225 mg m^−2^) plus concurrent radiotherapy. Patients had resectable OSCC. Resection was scheduled for 4–6 weeks after chemoradiotherapy. During phase I (dose escalation; *n*=19), weekly oxaliplatin 45 mg m^−2^ plus CI-5FU 225 mg m^−2^ was established as the MTD and was the recommended dosage for phase II. Oesophageal mucositis was the dose-limiting toxicity at higher doses. During phase II, histopathological responses (<10% residual tumour cells within the specimen) were observed in 10 of 16 patients (63%; 95% confidence interval: 39–82%). Overall, 16 of the 25 patients (64%) who underwent resection had a histopathological response; tumour-free resection (R0) was achieved in 80%. Neoadjuvant weekly oxaliplatin 45 mg m^−2^ plus CI-5FU 225 mg m^−2^ with concurrent radiotherapy provides promising histological response rates and R0 resection rates in locally advanced OSCC.

Although the best treatment strategy for locally advanced oesophageal squamous cell cancer (OSCC) is still being debated, the use of neoadjuvant chemoradiotherapy has gained acceptance (Gebski *et al*, 2007; Tepper *et al*, 2008). The rationale for chemoradiotherapy followed by surgery is potentially to downsize the tumour, thereby increasing the rate of tumour-free (R0) resections, reducing early relapses and improving survival. Overall, the impact of pre-operative treatment on survival depends on achieving a major pathological response at oesophagectomy (Swisher *et al*, 2005; Brücher *et al*, 2006). Pathological complete responses are associated with improved long-term survival but occur in only 20–40% of patients after pre-operative chemoradiotherapy (Bates *et al*, 1996; Walsh *et al*, 1996; Bosset *et al*, 1997; Ajani *et al*, 2001).

The most widely used pre-operative chemoradiotherapy combination – cisplatin and 5-fluorouracil (5FU) with concurrent radiotherapy – has failed to achieve a histopathological complete response rate above 30% or a long-term survival rate higher than 40% in patients with OSCC. Besides limited efficacy, this regimen is associated with substantial gastrointestinal toxicities, including nausea, mucositis and oesophagitis (Herskovic *et al*, 1992; Al-Sarraf *et al*, 1997). To enhance the efficacy and tolerability of multimodal treatment, new chemotherapeutic agents, such as oxaliplatin, have been incorporated into oesophageal cancer therapy.

Oxaliplatin is a potent radiosensitising agent both *in vitro* and in clinical practice (Blackstock *et al*, 1999; Cividalli *et al*, 2002; McMullen and Blackstock, 2002; Magne *et al*, 2003), and phase I and II trials suggest that it is at least as effective as cisplatin in OSCC whereas being better tolerated (Khushalani *et al*, 2002). If the goal of preoperative radiochemotherapy is to maximise tumour shrinkage before surgery, chemotherapy should be scheduled as dense as possible (that is, applied concomitantly and as often as possible during RT), to optimise local effects by sensitising tumour cells to radiation. The use of a weekly oxaliplatin schedule may, in addition, reduce acute toxicity and maximise the inhibition of sub-lethal radiation-induced DNA damage repair (Aschele *et al*, 2005). Addition of weekly oxaliplatin to continuous 5-FU or capecitabine and concomitant radiotherapy has been shown to be feasible and active in phase I and II studies in rectal cancer (Machiels *et al*, 2005; Rutten *et al*, 2006, Rödel *et al*, 2007). Moreover, the weekly application of oxaliplatin plus infusional 5 FU/folinic acid has also proven to be highly active in first-line metastatic colorectal (Porschen *et al*, 2007) and gastric cancer (Lordick *et al*, 2005). Of note, this regimen is also associated with an acceptable toxicity profile with a particularly low rate of sensory neuropathy (Grothey *et al*, 2002). Consequently, we conducted this study to define the maximum tolerated dose (MTD) of weekly oxaliplatin with continuous infusional 5FU (CI-5FU) plus concurrent radiotherapy in OSCC, and to assess the clinical activity at the recommended dose level (RDL).

## Materials and methods

This multicentre study was conducted at two institutions in Germany. The protocol was approved by the local ethics committee and the study was performed in accordance with the Declaration of Helsinki. All patients provided written informed consent.

In the phase I and II portion, patients (aged 18–70 years) with histologically confirmed, locally advanced, non-metastatic OSCC (cT2–4 N0–N+ M0) were eligible. All were candidates for curative surgery, with a Bartel score <21 (Bartels *et al*, 1998), no prior chemotherapy or radiotherapy and adequate organ function. Exclusion criteria included: oesophageal stent implantation, tracheobronchial tree invasion, tracheobronchial fistula, a second malignancy, uncontrolled infection, neuropathy grade >1 or congestive heart failure of New York Heart Association grade ⩾2.

### Study design

This was an open-label, non-randomised, phase I/II study. Initially, at least three patients were entered at each dose level (DL). Toxicities were recorded at each DL using the National Cancer Institute Common Toxicity Criteria version 2.0. A dose-limiting toxicity (DLT) was defined as any adverse event (AE) of grade ⩾3 clearly related to chemoradiotherapy. If no DLT was found, dose escalation was permitted. If any DLT occurred in the first three patients, three additional patients were treated at the same dose. If a DLT was then noted in one or more patients, no further dose escalation was permitted. The MTD was the highest dose at which fewer than two out of six patients experienced DLTs during chemoradiotherapy and/or in the immediate pre-operative phase. This DL was chosen as the RDL for phase II.

### Treatment plan

**Figure 1:**
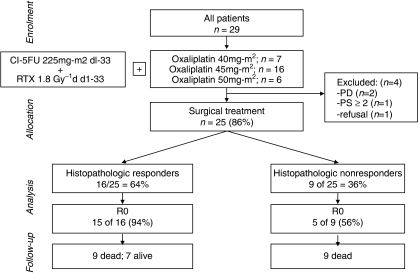
Flow diagram illustrating the study conduct and patient outcome according to histopathological response.

Chemotherapy consisted of escalating doses of weekly oxaliplatin (DL 1: 40 mg m^−2^; DL 2: 45 mg m^−2^; DL 3: 50 mg m^−2^ (Figure 1 Flow diagram); Supplementary Table A and Supplementary Figure A). Oxaliplatin was administered by intravenous infusion over 2 h every week for 5 weeks, and CI-5FU 225 mg m^−2^ was administered by 24-h continuous infusion on days 1–33.

Radiotherapy was administered for 5 days every week for 5 weeks at a dose of 1.8 Gy/day to the isocentre or a normalisation point that was representative of the target volume, up to a total dose of 45 Gy.

3D conformal external-beam radiotherapy with 6–15 MeV photons was delivered using three- and four-field techniques. The clinical target volume (CTV) comprised the primary tumour and adjacent lymph nodes with a margin of 4 cm in the craniocaudal direction. For cervical cancers the cervical lymph nodes up to the hyoid including the medial part of the paraclavicular lymph nodes had to be included. For tumours in the mid oesophagus lymph nodes in the upper anterior and posterior mediastinum were included in the CTV if they were suspicious for infiltration on CT and/or endosonography. For tumours in the lower third lymph nodes along the minor gastric curve and around the coeliac trunk were enclosed if they were suspicious on imaging.

The margin of the planning target volume to the CTV was 8–10 mm in all directions, taking internal organ movements as well as set up errors into account (Supplementary Table B).

Surgery was scheduled for 4–6 weeks after completion of chemoradiotherapy. Patients underwent standardised, transthoracic, en-bloc oesophagectomy with two-field lymphadenectomy (Siewert *et al*, 1998). Cervical tumours were treated with a partial oesophageal resection and reconstruction was carried out with a free jejunal graft (Steinau *et al*, 1988).

Patients were examined at 3, 6, 9 and 12 months and every 6 months thereafter. Assessment included physical examination, laboratory investigations, endoscopy, abdominal ultrasonography and computed tomography of the neck, chest and abdomen.

### Treatment-related morbidity and mortality

Any complication occurring after surgery was considered as postoperative morbidity, including clinically symptomatic anastomotic leakage, pulmonary, cardiovascular, infectious, and miscellaneous events. The overall postoperative mortality rate was defined as any death that occurred before a patient was discharged or even after discharge if there was any possible correlation with the operation itself.

### Histopathological work-up

Response to chemoradiotherapy was classified by quantification of residual tumour cells as follows: no residual tumour cells (complete regression, ypCR); <10% viable tumour cells (subtotal regression, ypSR); 10–50% viable tumour cells (partial regression, ypPR); >50% viable tumour cells (minimal regression) (Becker *et al*, 2003; Brücher *et al*, 2006). Histopathological response to radiochemotherapy was defined as <10% residual tumour cells, whereas histopathological non-response was defined as >10% residual tumour.

### Statistical analysis

The primary end point of the phase I part of the study was to define the MTD for oxaliplatin and CI-5FU plus radiotherapy; the primary objective of phase II was the histopathological response rate, defined as a complete or a subtotal histopathological response. The phase II study was designed as a Gehan two-stage trial (Gehan, 1961), assuming a response rate of ⩾50%. With a power of 95%, a sample size of five was required for the first stage. The sample size for the second stage was determined by the observed number of responses and the pre-specified precision of 10%.

Secondary study end points included clinical response rate, tolerability, completeness of tumour resection (R0 resection *vs* R1 or R2 resection), ypT and ypN categories, operative mortality and median overall survival (OS), event-free survival (EFS). OS and EFS were analysed in the intent-to-treat population and were calculated from the date of study assignment until death (OS) or until documented radiological or endoscopic progression, death or last contact (EFS). The probability of survival was estimated using the Kaplan–Meier method. As the response was defined by histopathological findings available only after surgery, event time analysis comparing histopathlogical responders and non-responders to neoadjuvant therapy was calculated from the day of surgery. Comparisons between patient groups were made by a log-rank test. The median survival and hazards ratio calculated by Cox's proportional hazards model were reported with 95% confidence intervals (CIs). Median follow-up time was calculated by the inverse Kaplan–Meier approach (Schemper and Smith, 1996). The Conditional Binomial Exact Test (Rice, 1988) was used to compare frequencies between patient groups and 95% confidence intervals for proportions were calculated according to Brown (Brown *et al*, 2001). All statistical analyses were performed at a 0.05 level of significance.

## Results

### Patient characteristics

From July 2003 to July 2005, 29 patients were enroled (Table 1). All patients received one or more doses of oxaliplatin and CI-5FU plus radiation, and were assessed for response, toxicity and survival.

### Dose escalation and DLTs

Seven patients were treated at DL 1. One of the first three patients experienced grade 3 mucositis, meeting the criteria for a DLT. Subsequently, four more patients were included, all of whom completed chemoradiotherapy with no DLTs. Owing to simultaneous inclusion of two patients at different study sites, an additional patient was enroled at DL 2; no DLTs were observed in the first three patients. Two of the six patients treated at DL 3 developed grade 3 mucositis needing temporary parenteral nutrition. Consequently, dose escalation was stopped and three additional patients were treated at DL 2, the RDL, one of whom developed grade 3 diarrhoea. Oxaliplatin 45 mg m^−2^ was therefore selected as the MTD. The RDL cohort was increased to 16 patients for the phase II study.

### Treatment delivery

The median duration of treatment was 5 (range, 3–5) weeks. Overall, 25 of 29 patients completed the planned treatment without interruption; the other four patients did not complete the treatment programme because of excessive toxicity.

### Toxicity

Haematological AEs were mild in the phase I (Supplementary Table C) and II studies (Table 2).

In phase I, one patient had grade 3 diarrhoea and three had grade 3 mucositis. One patient was hospitalised with toxic colitis and grade 3 diarrhoea. Two patients with oesophageal mucositis, treated at dose level 3, were hospitalised for intravenous hydration and nutrition.

### Histopathological response

The histopathological response rate (ypCR and ypSR) among all operated patients was 64% (16 of 25; 95%; CI: 43–82%). During phase II, histopathological responses were observed in 10 of 16 patients treated at the RDL (63%, 95% CI: 45–80%). It is interesting to note that two patients treated at the RDL could not be operated because of disease progression and were therefore considered histopathological non-responders.

### Clinical response

After chemoradiotherapy, the overall response rate according to RECIST was 45% (13 of 29 patients; 95% CI: 28.4–62.5%), including three complete responses (10%; 95% CI: 3.6–26.4%); 14 patients had stable disease (48%; 95% CI: 31.4–65.6%) and two patients had disease progression (7%; 95% CI: 1.9–22%). In the phase II population (*n*=16), eight patients (50%; 95% CI: 28–72%) achieved a response, including three complete remissions. In one patient (6%) the disease progressed during treatment (Supplementary Table D).

### Surgery

Overall, 25 patients (86%) underwent surgery 4–6 weeks after chemoradiotherapy. Four patients did not undergo surgery as a result of disease progression (*n*=2), deterioration in medical fitness (*n*=1) and patient refusal (*n*=1). Among the operated patients, 20 patients (80%) underwent R0 resection and five patients (20%) underwent R1 resection. No deaths occurred during surgery. The overall postoperative mortality rate was 12.0% (3 of 25 patients; one patient died because of a chylothorax, one because of bleeding, and one because of mediastinitis) (Table 3).

Histopathological responders had a significantly higher R0 resection rate (15 of 16 patients (94%); 95% CI: 71.6–98.8%) than non-responders (5 of 9 patients (56%); 95% CI: 26.7–81.1%; *P*=0.023).

### Survival

After a median follow-up of 33.5 months (range, 26.3–48.3 months) from date of study assignment, 7 of the 29 patients (24%) were still alive, with no evidence of disease recurrence in six patients (21%). Two patients developed locoregional, extraluminal recurrences, five had distant recurrences and seven had simultaneous locoregional and distant recurrences.

The median OS was 18 months (95% CI: 15.8–19.4 months; Supplementary Figure B), with an estimated 2-year survival rate of 28% (±8.3%).

The median EFS was 12.9 months (95% CI: 10.1–15.7 months; Supplementary Figure C), with a 2-year EFS rate of 24% (±7.9%).

**Figure 2:**
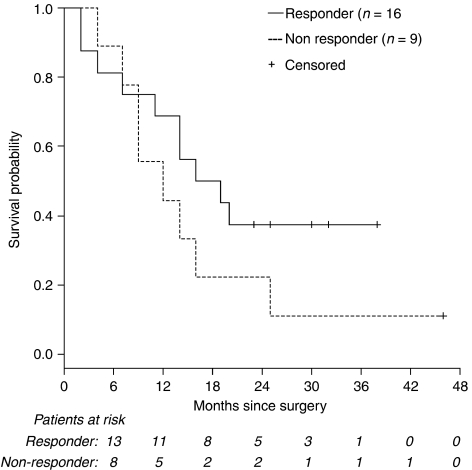
Survival by histopathological response (*P* (log-rank)=0.21 *vs* non-response). Survival is shown for all patients who underwent surgery (*n*=25). The median OS from date of surgery for histopathological responders was 16 months (95% confidence interval (CI): 6.2–25.8 months), respectively, compared with 12 months (95% CI: 3.2–20.8 months) in histopathological non-responders, hazard ratio: 1.78 (95% CI: 0.70–4.57).

**Figure 3:**
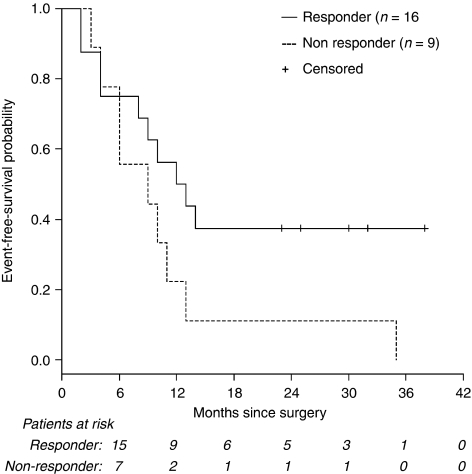
Event-free survival (EFS) by histopathological response (*P* (log-rank)=0.11). EFS is shown for all patients who underwent surgery (*n*=25). The median EFS from the date of surgery was 12 months in histopathological responders (95% confidence interval (CI): 6.1–17.9 months) *vs* 9 months for non-responders (95% CI: 0.3–17.8 months).

Comparison between histopathological responders (*n*=16) and non-responders (*n*=9) shows a non-significant trend towards a better OS (*P* (log-rank)=0.21 Figure 2) and EFS (*P* (log-rank)=0.11; Figure 3) in responding patients.

RO resection (*n*=20) was associated with a significantly improved OS (*P* (log-rank)=0.028) with a median OS of 16 months (95% CI: 8.7–23.3 months) *vs* 9.0 months in patients with incomplete resection (*n*=5; 95% CI: 6.8–11.1 months).

## Discussion

This phase I/II study demonstrated that a weekly regimen of neoadjuvant oxaliplatin 45 mg m^−2^, CI-5FU 225 mg m^−2^ per day plus concurrent radiotherapy is feasible and effective treatment for patients with locally advanced OSCC, with >50% of resected specimens showing major histopathological responses in the primary tumour.

One episode of grade 3 diarrhoea represented the only DLT at the RDL during the phase I dose-escalation study. Overall, clinical toxicities, particularly grade 3/4 haematological and gastrointestinal events, were less intense than previously reported with 5-FU- and cisplatin-based chemoradiotherapy (Bedenne *et al*, 2007). Toxicity assessment in patients treated at the RDL during phase II confirmed the excellent tolerability of the regimen, with mainly mild AEs and only a 6% overall incidence of grade 3 mucositis and diarrhoea (see Supplementary Table C). This rate of grade 3 mucositis did not exceed that reported previously for cisplatin-based chemoradiotherapy (Heath *et al*, 2000). Neurotoxicity, a potential concern because of the weekly oxaliplatin administration, consisted mainly of grade 1 events. These findings are comparable with other phase I/II studies using oxaliplatin-based chemoradiotherapy (Khushalani *et al*, 2002; Conroy *et al*, 2007). The favourable toxicity profile led to good compliance with treatment, and the full radiation course was delivered to all but two patients, and only four patients stopped chemotherapy because of toxicity.

However, a postoperative mortality rate of 12%, although it is in line with data from previous studies in this disease (Stahl *et al*, 1996, 2005; Van Lanschot *et al*, 2001) could reflect an important issue hampering satisfying long-term survival in this patient population.

The histopathological response rate of 64% in all operated patients (*n*=25) and the histopathological response rate of 63% observed during phase II (*n*=16), compare favourably with cisplatin-based neoadjuvant chemoradiotherapy (Bosset *et al*, 1997; Stahl *et al*, 2005; Brücher *et al*, 2006; Bedenne *et al*, 2007) and oxaliplatin-based chemoradiotherapy given every 2 weeks (Khushalani *et al*, 2002). This considerable activity may be a result of enhanced radiosensitisation achieved through weekly oxaliplatin administration. Pathological response rates across phase II and III trials vary significantly, so a randomised, comparative study would be required before definitive conclusions can be made regarding the superiority of this regimen over cisplatin-based regimens.

The high histopathological response rate observed would be expected to lead to a high R0 resection rate, which was 94% in the histopathological responders compared with 56% in the histopathological non-responders, confirming published data (Brücher *et al*, 2006). Complete resection, including negative microscopic margins, is a known positive prognostic factor in estimating the survival of patients undergoing oesophagectomy (Kelsen *et al*, 2007). The overall rate of 80% for R0 resections reported here in locally advanced disease compares favourably with previously reported rates (Hofstetter *et al*, 2002; Mariette *et al*, 2003).

Histopathological response, together with nodal status, appears to be the best predictor of outcomes after chemoradiotherapy (Swisher *et al*, 2005). However, although the median survival of 18 months was in the range reported previously, the 2-year survival rate of 28% observed here was suboptimal compared with survival rates reported for cisplatin-based chemoradiotherapy regimens in similar patient populations (Stahl *et al*, 2005; Bedenne *et al*, 2007). Moreover, better survival rates were reported with an oxaliplatin-containing chemoradiotherapy regimen in patients with inoperable oesophageal cancer (Conroy *et al*, 2007). A possible explanation for the relatively low observed 2-year survival rate could be the rather short-term neoadjuvant treatment duration of only 5 weeks in this trial. It is interesting to note that compared with The French Multicenter Study (Bedenne *et al*, 2007) and the German trial (Stahl *et al*, 2005) no induction chemotherapy before the start of combined radiochemotherapy was administered. This indicates that, although the current study included a thoroughly staged and uniformly defined population, the optimal treatment for patients presenting with locally advanced OSCC has not yet been defined. The high number of distant relapses may have led to relatively low survival rates and suggests that further refinement of the chemotherapy regimen is warranted. Therefore, intensification of the treatment regimen, for example, by performing a sequential application of a high-dose induction chemotherapy followed by radiochemotherapy, as investigated by Stahl *et al* (2005) could provide a better control of systemic disease. Also, addition of other chemotherapy drugs like taxanes may add some additional effectiveness (Meluch *et al*, 2003; van de Schoot *et al*, 2008).

Overall, neoadjuvant chemoradiotherapy for locally advanced OSCC may improve histopathological response and R0 resection rates. Nevertheless, the impact of additional surgery on survival is still being debated, with recent phase III trials failing to demonstrate a consistent survival benefit over chemoradiotherapy alone (Stahl *et al*, 2005; Bedenne *et al*, 2007).

In conclusion, weekly oxaliplatin, CI-5 FU and conventionally fractionated radiotherapy proved a feasible regimen with a promising pathological response rate and a high R0 resection rate in a uniform and well-staged group of patients with locally advanced OSCC. However, due to a high distant relapse rate, the 2-year survival rate in this patient population was rather disappointing.

To optimise treatment further, new targeted agents combined with conventional cytotoxic therapy are being evaluated. In particular, the activity of a weekly regimen of oxaliplatin plus CI-5 FU and radiotherapy with the addition of the epidermal growth factor receptor inhibitor, cetuximab is currently being investigated in a prospective phase II trial in locally advanced OSCC.

## Figures and Tables

**Table 1:** Patient and tumour characteristics

**Characteristics**	**No. of patients (*n*=29)**	**%**
*Age (years)*		
Median	60	
Range	19–67	
		
*Sex*		
Male	25	86
Female	4	14
		
*ECOG performance status*		
0	21	72
1	8	28
		
*Clinical stage* a		
uT2	2	7
uT3	26	90
uT4	1	3
uN+	29	100
		
*Differentiation*		
Well differentiated (G1)	0	0
Moderately differentiated (G2)	10	35
Poorly differentiated (G3)	18	62
Undifferentiated (G4)	1	3
		
*Tumour location*		
Cervical oesophagus	6	21
Mid thoracic	11	38
Lower thoracic	12	41

ECOG=Eastern Cooperative Oncology Group.

a As determined by endoscopic ultrasonography.

**Table 2:** Haematological and non-haematological toxicities (National Cancer Institute Common Toxicity Criteria, version 2.0) observed in the phase II study

	**No. of patients (*n*=16)**				
**Toxicity grade**	**1**	**2**	**3**	**4**	**Total (%)**
*Haematological toxicity*					
Anaemia	9	1	—	—	10 (63)
Neutropenia	4	1	—	—	5 (31)
Febrile neutropenia	—	—	—	—	—
Thrombocytopenia	3	—	—	—	3 (19)
					
*Non-haematological toxicity*					
Diarrhoea	5	1	1	—	8 (50)
Nausea	6	1	1	—	8 (50)
Emesis	7	2	—	—	9 (56)
Mucositis within RT field	5	3	1	—	9 (56)
Mucositis outside RT field	1	—	—	—	1 (6)
Sensory neuropathy	6	2	—	—	8 (50)
Cold-related dysaesthesias	2	1	—	—	3 (19)
Hand-foot syndrome	—	—	1	—	1 (6)
Lethargy	10	3	—	—	13 (81)

RT=radiotherapy.

**Table 3:** Surgical outcome, post-operative morbidity and mortality (*n*=25)

**Variable**	**No. of patients**	**%**
Total resections	25	100
		
*Type of resection*		
R0	20	80
R1	5	20
		
*Total morbidity*	23	92
Anastomotic leakage	12	48
Pulmonary complications	5	20
Necrosis of intestinal interponate	2	8
		
Bleeding	2	8
Chylothorax	1	4
Retrosternal abscess	1	4
Pleural empyema	1	4
Gastroparesis	1	4
		
Overall postoperative mortality rate	3	12
