# Supplementary material for: Neoadjuvant continuous infusion of weekly 5-fluorouracil and escalating doses of oxaliplatin plus concurrent radiation in locally advanced oesophageal squamous cell carcinoma: results of a phase I/II trial
Source: Br J Cancer. 2008 Sep 16;99(7):1020–6. doi: 10.1038/sj.bjc.6604659 (PMC2567089; doi:10.1038/sj.bjc.6604659)
Supplement: Supplementary Data [file 6604659x1.doc]

Supplementary data

**Neoadjuvant continuous infusion of weekly 5-fluorouracil and escalating doses of oxaliplatin plus concurrent radiation in locally advanced oesophageal squamous cell carcinoma: results of a phase I/II trial**

S. Lorenzen^1^, B. Brücher^2^, F. Zimmermann^3^, H. Geinitz^3^, J. Riera^4^, T. Schuster^5^, N. Roethling^6^, H. Höfler^7^, K.Ott^8^, C. Peschel^1^, J. R. Siewert^9^, M. Molls^3^ & F. Lordick^10^

*^1^Third Department of Internal Medicine (Haematology/Medical Oncology), Klinikum rechts der Isar, Technical University of Munich, Munich, Germany; ^2^Department of Surgery, University of Tübingen, Tübingen, Germany; ^3^Department of Radiation Oncology, Klinikum rechts der Isar, Technical University of Munich, Munich, Germany; ^4^University Hospital Gießen-Marburg GmbH, Marburg, Germany; ^5^Institute of Medical Statistics and Epidemiology, Munich, Germany; ^6^Munich Center for Clinical Studies, Munich, Germany; ^7^Institute of Pathology, Munich, Germany; ^8^Department of Surgery, University of Heidelberg, Heidelberg, Germany; ^9^Board of Directors of the University Hospital, University of Heidelberg, Heidelberg, Germany; ^10^Department of Medical Oncology, National Centre for Tumour Diseases, University of Heidelberg, Heidelberg, Germany.*

**MATERIALS AND METHODS**

**Pretreatment evaluation**

All patients underwent a complete medical history, physical examination, full blood count, biochemical profile and electrocardiography. Tumour staging included computed tomography (CT), gastro-oesophageal endoscopy, endoluminal ultrasonography and bronchoscopy (in case of tumour localisation adjacent to the tracheobronchial tree).

Pre-treatment laboratory tests inclusion criteria included: a total leucocyte count ≥3000/μl, absolute neutrophil count ≥2000/μl, thrombocyte count ≥100 000/μl, total bilirubin ≤1.5 x upper limit of normal (ULN), transaminases ≤2 x ULN and creatinine clearance >70 ml/min.

Patients attended weekly for the clinical examination, blood counts and biochemical profile. All staging work was to have been completed within the 2 weeks before treatment.

## Treatment

The study design is shown in Figure A and the dose-escalation scheme in Table A. All patients received standard anti-emetic prophylaxis of a 5-hydroxytryptamine-3 receptor antagonist plus dexamethasone. If the neutrophil count was <1200/µl and the platelet count <75 000/µl, chemotherapy was postponed until recovery. Chemotherapy was then resumed with a dose reduction of 20% for either drug. Chemotherapy was discontinued for any grade 3/4 non-haematological toxicity other than grade 3 skin reaction and mucositis/oesophagitis over the irradiated area. The dose of oxaliplatin was reduced by 20% if grade 3 peripheral neuropathy occurred.

Patients underwent pre-operative re-staging by CT and endoscopy. If there was evidence of distant metastasis before surgery, the patient was removed from the protocol-specified treatment plan and offered palliative care.

## RESULTS

## Toxicity

Toxicities observed in the phase I study are shown in Table C. At dose level (DL) 1, one patient developed grade 3 mucositis and another had an allergic reaction to oxaliplatin; one patient had grade 3 diarrhoea at DL 2; two patients experienced grade 3 mucositis at DL 3. Dose modifications for chemotherapy according to the protocol guidelines were performed only in the patient with grade 3 diarrhoea.

Of the 26 patients who received the scheduled 45 Gy radiation dose, radiation dose modifications were required in three patients treated at DL 2. This was necessary because of toxicity-related early discontinuation of treatment in two patients and disease progression rendering surgery of curative intent impossible in a third.

## Downstaging

Nodal downstaging (cN+ to ypN0) was seen in 13 of 25 patients (52%) and the T staging was scaled down in 17 of 25 patients (68%) (Table D). On examination of the resected specimen, the histopathological response evaluation revealed complete tumour regression (ypCR) in 6 of 25 patients (24%). Overall, subtotal regression (ypSR) was observed in 10 patients, partial regression (ypPR) in 7 and minimal regression in 2.

## Surgery

Surgery was dictated by the location of the tumour. Six of the 25 patients (24%) underwent partial resection of the proximal oesophagus and 19 (76%) transthoracic subtotal oesophagectomy.

Univariate analysis revealed that histopathological responders had a significant shift towards more favourable T staging (14/16; 88%) versus nonresponders (2/9; 22%;
*P*= 0.002). However, a shift in N staging was not significantly different in the two groups (responders 10/16 [63%] versus nonresponders 3/9 [33%]; *P* = 0.226).

## Survival

Kaplan–Meier plots for OS and EFS are shown in Figures B and C. The estimated 2-year survival rate and the median OS for histopathological responders were 37.5% (± 12.1%) and 18.7 (95% CI: 10.6–26.7) months, respectively, compared with 22.2% (± 13.9%) and
15.2 months (95% CI: 10.5–19.8 months; *P* = 0.227) in histopathological nonresponders (hazard ratio: 1.77; 95% CI: 0.70–4.53).

**Reference**

1. Sobin LH (2003) TNM, sixth edition: New developments in general concepts and rules. *Semin Surg Oncol*  **21**: 19–22

**Table A. Dose-escalation scheme**

| **Dose level** | **Oxaliplatin (mg/m^2^)** | **CI-5FU (mg/m^2^/day)** | **RT (Gy/day x 25)** |
| --- | --- | --- | --- |
| 1 | 40 | 225 | 1.8 |
| 2 | 45 | 225 | 1.8 |
| 3 | 50 | 225 | 1.8 |

CI-5FU, continuous infusion 5-fluorouracil; RT, radiotherapy.

**Table B.** Maximum tolerated doses (MTDs) for organs at risk

| **Organ** |  | **MTD (Gy) in** |  |
| --- | --- | --- | --- |
|  | **100% of organ** | **66% of organ** | **33% of organ** |
| Lung | 12 | 15 | 25 |
| Spinal cord | 43 | 43 | 43 |
| Heart | 32 | 36 | 45 |
| Stomach | 20 | 32 | 40 |
| Liver | 20 | 25 | 40 |
| Kidney | 15 | 18 | 21 |

**Table C. Haematological and non-haematological toxicities (National Cancer Institute Common Toxicity Criteria, version 2.0) in the phase I study according to DL (cohorts 1–3)**

| **Toxicity grade** | **Cohort 1 (*n* = 7) at DL1** | | | | **Cohort 2 (*n* = 6) at DL2** | | | | **Cohort 3 (*n* = 6) at DL3** | | | |
| --- | --- | --- | --- | --- | --- | --- | --- | --- | --- | --- | --- | --- |
|  | **1** | **2** | **3** | **4** | **1** | **2** | **3** | **4** | **1** | **2** | **3** | **4** |
| *Haematological toxicities (n)* Anaemia Neutropenia Febrile neutropenia Thrombocytopenia | 5 2 — — | — 1 — — | — — — — | — — — — | 3 0 — — | — — — — | — — — — | — — — — | 5 1 — 1 | — — — 2 | — — — — | — — — — |
| *Non-haematological toxicities (n)* Diarrhoea Nausea Emesis Mucositis within RT field Mucositis outside RT field Sensory neuropathy Cold-related dysaesthesias Hand–foot syndrome Lethargy | 1 1 1 1 — 1 — — 2 | — — — 1 — — — — — | — — — 1 — — — — — | — — — — — — — — — | 2 3 3 3 — 2 — — 2 | 1 1 1 2 — 2 — — 2 | 1 — — — — — — — — | — — — — — — — — — | 1 4 2 2 — 3 — 2 4 | — — — 1 — 1 — — 1 | — — — 2 — — — — — | — — — — — — — — — |

DL: dose level; RT: radiotherapy.

**Table D. Pathological stage (ypT, ypN) compared with clinical stage (uT, cN) at baseline in patients undergoing tumour resection (*n*= 25^a^)**

| **Clinical stage** |  | **Pathological stage** | | | | |
| --- | --- | --- | --- | --- | --- | --- |
|  | **n** | **ypT0** | **ypT1** | **ypT2** | **ypT3** | **ypN1** |
| uT2 | 2 | — | 2 | — | — | — |
| uT3 | 22 | 6 | 2 | 5 | 9 | — |
| uT4 | 1 | — | — | 1 | — | — |
| Total | 25 | 6 | 4 | 6 | 9 | — |
| cN1 | 25 | — | — | — | — | 12 |

^a^Four patients did not undergo resection.

**Figure legends**

**Figure. A** Study design. CI-5FU: continuous infusion 5-fluorouracil; CTx: chemotherapy; IV: intravenous; RCTx: radiochemotherapy; RTx: radiotherapy.

**Figure. B** Kaplan–Meier estimates of overall survival from the date of randomisation in the intention-to-treat population (*n* = 29).

**Figure. C** Kaplan–Meier estimates of median event-free survival from the date of randomisation in the intention-to-treat population (*n* = 29).

Figure A

**RTx**

**d1**

**d33**

**1.8 Gy/day; total dose 45 Gy**

**CTx**

**d1**

**CI-5FU 225 mg/m^2^/day**

**Oxaliplatin 40–50 mg/m^2^ once weekly 2-hour iv infusion (3 cohorts)**

**d8**

**d15**

**d22**

**d29**

**Resection**

**4–6 weeks**

**post-RCTx**

Figure B

*Patients at risk:*

*29 26 21 14 8 4 3 1 0*

Figure C

*Patients at risk:*

*29 23 17 8 7 4 3 0*
